# Alternative Plant Protection Strategies Using Bacteria and Thyme to Improve Strawberry (cv. Elsanta) Yield and Quality

**DOI:** 10.3390/plants14121827

**Published:** 2025-06-14

**Authors:** Neringa Rasiukevičiūtė, Armina Morkeliūnė, Ingrida Mažeikienė, Juozas Lanauskas, Alma Valiuškaitė

**Affiliations:** 1 Laboratory of Plant Protection, Institute of Horticulture, Lithuanian Research Centre for Agriculture and Forestry, Kaunas District, Babtai LT-54333, Lithuania; armina.morkeliune@lammc.lt (A.M.); alma.valiuskaite@lammc.lt (A.V.); 2 Department of Orchard Plant Genetics and Biotechnology, Institute of Horticulture, Lithuanian Research Centre for Agriculture and Forestry, Kaunas District, Babtai, LT-54333, Lithuania; ingrida.mazeikiene@lammc.lt; 3 Department of Horticulture Technologies, Institute of Horticulture, Lithuanian Research Centre for Agriculture and Forestry, Kaunas District, Babtai LT-54333, Lithuania; juozas.lanauskas@lammc.lt

**Keywords:** strawberry quality, *Bacillus halotolerans*, *Bacillus velezensis*, thyme, alternative plant protection

## Abstract

Alternative plant protection methods should be promoted to mitigate the dangers and consequences of using chemical pesticides, ensuring a safe environment and protecting human health (Directive 2009/128/EC). One of the objectives of the EU organic production action plan is to provide substitutes for plant protection methods, decrease the adverse effects on the environment, and promote the diversity of living organisms. The use of synthetic and non-organic chemicals has significantly expanded, damaging human health and the environment. This study aimed to evaluate alternative plant protection solutions for the improvement of the strawberry cv. Elsanta plant’s generative development, yield, fruit quality, and biochemical composition. The two-year strawberry experiment conducted in a tunnel greenhouse included chemical and biological means (Bacteria and Thyme preparations). The experiment randomised a block design with four replicates and 32 plants per replicate. The treatments were conducted at the 10% flowering state (BBCH 61–65), every 7–10 days (a total of four times): (1) Control, (2) Chemical, (3) Bacteria, and (4) Thyme. We evaluated the yield, fruit weight, size, number of leaves, crowns, flowers, inflorescences, fruit firmness, soluble solids, and Vitamin C. The highest fruit weight at the first picking was in the Bacteria treatment. The number of rotten fruits was similar after all treatments. Additionally, they were firmer and bigger in size but had a smaller soluble solids content. The strawberry ascorbic acid and soluble solids content (Brix %) showed significant variation. The highest ascorbic acid concentration in the fruit was after the Thyme application (45.06%). Our study showed that alternative plant protection measures can reduce the use of chemical fungicides and maintain proper fruit quality.

## 1. Introduction

The European Commission aims to achieve its objectives by using sustainable pesticides and promoting organic farming by 2030. Promoting alternative plant protection methods is necessary to mitigate the hazards and consequences associated with chemical pesticides, hence ensuring a safe environment and safeguarding human health (Directive 2009/128/EC). One of the objectives of the EU organic production action plan is to create substitutes for plant protection methods, minimise the adverse effects on the environment, and promote biodiversity [1].

Strawberries are highly cultivated and favoured fruits because of their abundant antioxidants and pleasant taste [1,2]. The management of strawberry (*Fragaria × ananassa* Duch.) diseases has relied on chemical control methods. However, due to the expense of pesticides and their negative environmental impact, it is crucial to accurately establish the appropriate period for their application. The excessive usage of agricultural chemicals adversely affects soil quality and is a significant barrier to enhancing the productivity of crops. It also contaminates air, water, and soil, leading to severe health issues [3]. Strawberries are highly perishable and require particularly rigorous postharvest management. The fruit becomes more susceptible to degradation and postharvest infections during storage, changing its biochemical and physiological features. Consumers appreciate the high quality of strawberries, value their production under sustainable conditions, and focus on cost-effective cultivation [2,3]. Various postharvest procedures, including spraying, coating, or dipping, help to preserve the quality. Various horticultural plants are injured by diseases caused by *Botrytis* [4]. They are primarily caused by biological factors that determine the interaction between the host plant, natural conditions, and chemical and physical processes [4,5,6]. Strawberries’ principal commercial value is their flavour, which is influenced by sugars and acids in ripe fruits [2].

*Botrytis* is one of nature’s most widely distributed pathogens, causing significant yield losses of fruits, berries, vegetables, and ornamental plants during vegetation and storage. This fungus is responsible for causing around 80–85% of strawberry losses, particularly under conditions conducive to disease development [5,6,7]. The range of the pathogen is very wide, affecting plants from the southern to the northern hemisphere, including plants growing in extremely cold regions or deserts [5]. Climate change promotes the spread of plant diseases, and the disease development becomes difficult to predict. Fluctuations in the precipitation, temperature, and leaf moisture affect plant disease [8].

The increased interest in natural, non-chemical plant protection encouraged researchers to look for alternative means. Applying new natural active substances for plant protection can solve pesticide resistance problems and reduce environmental and food pollution [9]. However, most research is linked only to plant disease control by essential oils, not to the plant generative organs, yield, or fruit quality. Plant protection can be achieved by utilising natural antibacterial compounds as a substitute for chemical substances. Plant-based plant protection agents, such as essential oils and extracts, are gaining much interest and are being actively explored by scientists worldwide as an alternative to chemical fungicides [10]. Essential oils (EOs) and their products are primarily used in food and medicine. EO is a novel and cutting-edge field that enhances the microbiological safety of horticultural crop production [11]. The EO extracted from plants is pure oil, the constituents of which are terpenes, terpenoids, and aliphatic and aromatic compounds. Their most important feature is that they contain biologically active compounds and antioxidants that have an antimicrobial effect on strawberry pathogens [12,13,14,15]. EOs are often characterised as secondary metabolites with a high plant protection effect due to their antimicrobial properties, being biodegradable, and being non-toxic [12,13,14,15,16,17,18]. The EO of sage inhibits *Fusarium* spp. growth, peppermint EO inhibits *Alternaria* spp. and *Fusarium* spp. spread of pathogens [19]. Pathogens controlled by medicinal Thyme EO are *Monilinia fructicola*, *Botrytis cinerea*, and *Aspergillus flavus* [20,21]. Aćimović et al. [22] found an inhibitory effect of 90% of calendula EO on colony growth at higher concentrations. Mandal and Mandal [23] investigated EO concentrations of 1500–2000 µg mL^−1^ of calendula, which are effective against *Alternaria* spp., *Fusarium* spp., and other pathogens.

Plant-associated microorganisms are essential in maintaining a healthy physiological state of the host plant, and a reduced microbial diversity often affects the development of plant diseases. The diversity of Bacteria is abundant. Bacterial control agents as sustainable alternatives to chemical treatments are effective in many cases [24,25]. Natural antagonistic microorganisms can control a large number of plant pathogenic diseases. Some Bacteria suppress *Fusarium* spp., *Bipolaris* spp., and *Verticillium* spp. pathogens [26,27]. Among these, *Bacillus* species have emerged as promising candidates due to their broad-spectrum antifungal activity, rapid colonisation ability, and resilience in diverse environmental conditions. Members of the *Bacillus* genus produce a variety of bioactive compounds, including lipopeptides, enzymes, and volatile organic compounds (VOCs), which can inhibit fungal growth, disrupt spore germination, and induce systemic resistance in host plants [28]. Moreover, their capacity to form endospores enhances their survivability and shelf life, making them suitable for commercial formulations [29].

Thyme essential oil and *Bacillus* spp. Bacteria are potential alternative plant protection agents. The effects of alternative plant protection measures on various horticultural plants were collected from previous studies [10,14,30,31,32,33,34]. In our previous studies, we have identified the composition of Thyme essential oil. We have also found that it has antifungal effects against various plant pathogens, including strawberries. It enhances EOs to promote the strawberry root system in vitro and enhance vegetative growth. Additionally, EO promotes the increase in vitamins, anthocyanins, and phenolic contents in plants [35]. Beneficial microorganisms, which include biopesticides and biostimulants, provide an alternate approach to management. According to Soppelsa et al. [36], alfalfa hydrolysate, vitamins, chitosan, and silicon effectively enhanced strawberry growth in nutrient-deficient conditions. Biopesticides, including *Ampelomyces quisqualis, Bacillus subtilis*, and *Trichoderma harzianum* T39, decreased the growth of strawberry mildew [37]. The growth of strawberry powdery mildew was notably lower due to a strain of *Bacillus cereus* [38] and a strain of *B. amyloliquefaciens* [39]. Beneficial microorganisms from Bacteria mitigate drought and salt stress and promote plant growth [40]. *Bacillus* spp. produces antifungal biomolecules, chitinase, and glucanase. Additionally, *Bacillus* spp. improve the nutrient transport and photosynthetic rate, resulting in a higher growth rate and yield [41].

The current objectives worldwide are focused on achieving food security using sustainable methods and fewer chemical pesticides. A healthy plant is essential to obtaining a high-quality strawberry yield. This study evaluated and compared alternative vs. chemical plant protection solutions for improving the strawberry plant generative organs (leaves, crowns, flowers, and inflorescences), yield, fruit quality (weight, size, and firmness), and chemical composition (ascorbic acid soluble solids).

## 2. Results and Discussion

The inadequate effectiveness of fungicides, their adverse impact on human health and the environment, and public and regulatory concerns regarding the presence of fungicide residues in strawberry cv. Elsanta berries have highlighted the necessity of identifying alternative treatments to manage plant diseases [32,33,42,43].

The wide use of EO for plant protection increased the demand for research focused on evaluating yield and improving its quality. Researchers are investigating alternative treatments to promote plant development, manage pests, and achieve sustainable agriculture. Using synthetic fertilisers and pesticides in agricultural production has resulted in environmental and health risks [35,44]. Some strains of *Pseudomonas fluorescens* d17, e12, and *P. jesseneie* e39 increased biometric parameters, such as the leaf growth rate, and *Agrobacterium rubi* m39 increased the number of fruits [40]. Measuring the amounts of reproductive and generative organs of strawberry plants is essential. The number of crowns is directly related to the fruit yield [45]. The usage of EO improved the strawberry cv. Festival yield and increased vegetative growth. The stimulating EO plant growth characteristics could be due to a high nutrient uptake or root improvement. In addition, some EOs contain phenols with plant growth promotion characteristics [35].

### 2.1. Plant Vegetative and Generative Development

The average data on strawberry vegetative and generative organ parameters revealed that Bacteria slightly impact the strawberry plant (Table 1). The strawberry crown, leaves, runners, and inflorescence varied within the error range. However, in 2023, Bacteria treatments had the highest number of crowns, flowers, and inflorescences.

In 2024, there was a slight effect on Bacteria, but it is still unclear because there were only a few applications. The number of crowns, flowers, and leaves produced by Bacteria was higher than in other treatments. Our two-year mean data of strawberry generative organ parameters showed that, in most cases, the measures used had a tendency to only increase the number of crowns, leaves, flowers, and inflorescences.

### 2.2. Yield

The data revealed that during the study, the total yield of strawberries in the Chemical treatment was the lowest compared to others (a total amount of 7264.98 g) (Table 2). Strawberries treated by Bacteria produced the highest total yield, 9583.57 g. In addition, strawberries treated with Thyme were healthier than other treatments. It should be noted that the alternative plant protection products used in this study were more effective than the chemical plant protection products in 2023. The yield of strawberries was higher in 2024 than in 2023. Strawberries treated with the Chemical were healthier than other treatments. However, the highest yield was determined in the treatments with alternative plant protection products (17,794.04 and 18,140.23 g) compared to the Chemical (16,695.02 g). Similarly to our research, *B. velezensis* CE 100 increased the fruit yield and enhanced plant growth [41].

### 2.3. Average Fruit Weight and Size

The large fruit size usually correlates with average fruit weight and leads to a higher yield [2]. The fruit’s size also correlates with the size of the blooms and is influenced by temperature conditions.

Strawberries treated with Bacteria had the significantly highest weight (8.19 g) at the time of the first picking (Table 3). The results of the Chemical and Thyme were similar (7.05–7.39 g). The lowest weight was in the Control. However, there were no significant differences between the Chemical and Thyme treatments. During the second picking, the weight of strawberries in the Control remained almost unchanged (6.73 g). Strawberries treated with the Chemical had a significantly higher weight (8.28 g) than other treatments (6.34–7.35 g). The weight decrease was observed at the third picking in all treatments. The most notable differences were observed between the Control (5.15 g) and Chemical (7.14 g). No significant differences were recorded between strawberries treated with Bacteria and Thyme in 2023. The weight of strawberries in 2024 was higher than in 2023. The results for the Chemical and Thyme were comparable (12.97–12.60 g). The lowest weight was recorded in the Control (11.44 g). Nonetheless, no substantial changes were seen between the Chemical and Thyme treatments in the first picking. During the second picking, the weight of strawberries in the Control dropped to 9.78 g. Strawberries treated with Thyme had a higher weight (15.53 g) than other treatments (10.42–10.72 g). A weight reduction was seen on the third harvest across all treatments. No significant changes were observed between treatments.

The size of strawberries varied during the first and third picking in 2023 (Table 4). Strawberries treated with Bacteria had significantly higher diameters (2675 mm) at the time of the first picking. Strawberries treated with the Chemical and Thyme were similar (25.68–25.30 mm). Compared to the Chemical and Thyme treatments, the significantly lower-size strawberries were in the Control, with a diameter of 23.78 mm. In addition, the size of the strawberries increased in all treatments (Table 4). However, the treatment with Thyme slightly decreased the size of the strawberries (24.53 mm) compared to the Control. No significant differences were recorded in the Control, Bacteria, and Thyme treatments during the third picking. Weight loss is attributed to the respiration and water loss induced by transpiration. Thus, the primary cause of the undesirable impact on the shelf life of strawberries is the fast depletion of water through the skin [46]. The size of the strawberries was stable during the first and second picking between all treatments. However, the size of the strawberries decreased in the third picking in 2024. The size of the Bacteria treatments was slightly higher in all treatments. It should be noted that the total size of strawberries was higher than in 2023.

### 2.4. Strawberry Fruit Firmness

The strawberry firmness was measured every picking time from 2023 to 2024 (Table 5). According to the results, with an increasing storage time, the amount of fruit tissue firmness varies in all treatments. Tissue firmness is one of the most important physical parameters to evaluate fruit quality in the ripening and storage stages. The structural cell wall alterations, such as the breakdown of hemicellulose and galactose and the disintegration of pectin, cause tissue softness. It is also enzymatically mediated by the cell wall hydrolysis [46,47,48]. Strawberries treated with Bacteria were firmer than the other treatments (9.84 N cm^−2^). Strawberries treated with the Chemical were firmest during the second and third measurements (12.07–12.81 N cm^−2^, respectively). A significantly lower result was observed with Thyme (10.92 N cm^−2^) in 2023. Strawberries were firmer at the first picking in 2024 compared to 2023. In the second picking, the firmness was consistent with that observed in 2023. During the third picking, it was observed that the firmest strawberries were present in the Control (15.31) N cm^−2^ treatment.

### 2.5. Soluble Solids

The soluble solids of strawberries were measured thrice in 2023–2024 (Table 6). Strawberries treated with Bacteria had a smaller soluble solid content (8.45%) at the first measurement. Results observed in the Control, Chemical, and Thyme were similar (9.23–9.40%). During the second measurement, the highest soluble solids content was in the Control (11.25%). Meanwhile, the results of other treatments were similar (10.43–10.70%). The most notable differences were observed between the Chemical and Thyme (9.50–11.40%) at the third measurement. No significant differences were recorded between strawberries treated with Bacteria and those in the Control in 2023. The soluble solids concentration was lower in 2024 than in 2023. At the first measurement, the Control had a lower soluble solids content in strawberries, recorded at 6.69%. The results obtained in the Chemical, Bacteria, and Thyme were similar, ranging from 7.36% to 7.99%. In the second measurement, the Control had the highest soluble solids level at 8.11%. Concurrently, the outcomes of other treatments were comparable (6.75–7.05%). The Chemical and Thyme detected the most significant variations (6.68–8.39%) in the third assessment. No notable variations were seen between strawberries subjected to the Bacteria treatment and those subjected to the Control treatment. The highest soluble solids concentration was in the lavender EO treatment with a 5 mL/l concentration. The Thyme EO positively affects the fruit quality, with the increased soluble solids of grapes [35].

### 2.6. Content of Ascorbic Acid

**Figure 1:**
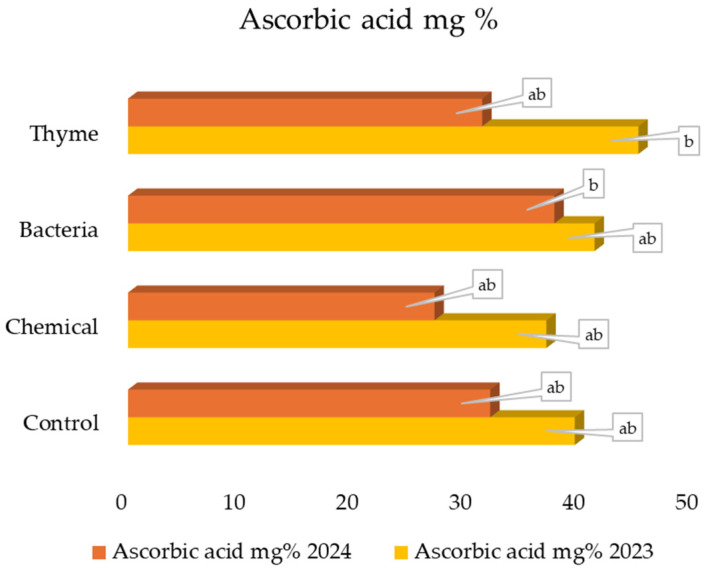
The strawberry fruit’s content of ascorbic acid after different alternative plant protection treatments, mg%. According to Duncan’s test (*p* < 0.05), the same letter indicates no significant differences between treatments.

Ascorbic acid is highly susceptible to decomposition in storage compared to other nutrients, mainly because of oxidation [49]. In 2023, Thyme had the highest concentration of ascorbic acid (45.06%) (Figure 1), and the results were significant compared to other treatments. The Chemical contained the lowest amount (36.94%). Bacteria had the highest concentration of ascorbic acid (37.65%), and the results were significant compared to other treatments in 2024. Chemical contained the lowest amount (27.06%). The present study shows that EO and Bacteria increased the ascorbic acid concentration, similarly to Shala et al. [35]. Shala et al. observed that the lavender and citronella EO, after applications at high concentrations, increased strawberry fruits’ ascorbic acid, phenols, and anthocyanins [35]. Possibly, the ascorbic acid (per fresh weight) was higher in the larger fruit.

Strawberries met the first-class requirements of the first–second picking, and the equivalent was larger than 25 mm in diameter. The largest strawberry diameter was determined during the first picking of the Thyme and Chemical treatments. Biostimulants based on essential oils and plant extracts, biologically active molecules, can influence the plant growth and development by influencing the metabolism and other processes [33,36].

Due to the high need for disease control, alternative treatments are frequently suggested alongside chemical agents as a control technique. All examined strains of *Bacillus* spp. in this study possess plant-growth-promoting attributes, which may indirectly bolster plants’ resistance to infections. Enhanced plant health and vitality make the plant less vulnerable to illnesses. These genes participate in nutrient absorption, stress resilience, phytohormone regulation, signal generation, and the enhancement of plant development. Several investigations have demonstrated that employing microorganisms with antagonistic or resistance-inducing capabilities is viable for preventing strawberry disease [50].

Our study showed that effectively incorporating alternative plant protection treatments makes achieving greater quality strawberries and reducing chemical fungicide usage possible. This reduction in fungicide use helps to prevent the development of a resistance to fungicides, hence delaying the emergence of fungicide resistance and prolonging the effectiveness of fungicides. Essential oils and Bacteria display a biostimulating effect on the strawberry plant’s organs, yield, and fruit quality. The approach will be explored in future studies, and it is also essential to investigate how *Botrytis cinerea* will act in alternative plant protection treatments.

## 3. Materials and Methods

### 3.1. Plant Material

**Figure 2:**
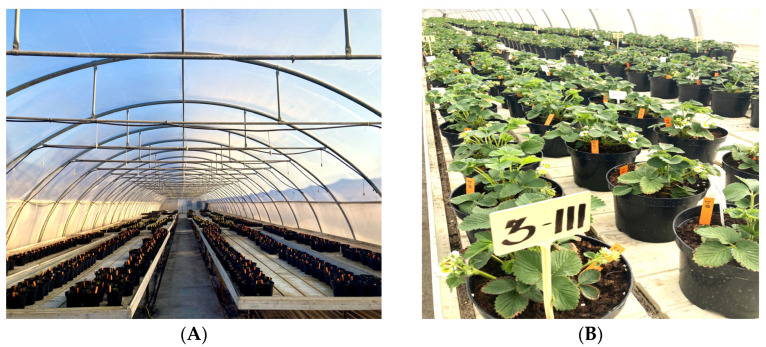
Tunnel greenhouse and strawberries cv. Elsanta planted in plastic pots. (**A**) Tunnel greenhouse and (**B**) strawberry plants in pots.

Strawberries cv. Elsanta were cultivated in a tunnel greenhouse in plastic pots with a capacity of 3 L (dimensions of 19 × 15 cm), Figure 2. The pots were filled with peat substrate (Terraerden, Rucava, Latvia). The declared mineral nutrients in the substrate were nitrogen (N) 140–210 mg L^−1^, phosphorus (P_2_O_5_) 160–240 mg L^−1^, and potassium (K_2_O) 180–270 mg L^−1^. Substrate also contained microelements such as Mn, Cu, Mo, B, Zn, and Fe. The pH, when measured in water, was 5.5–6.5, and the electrical conductivity (EC) was less than 1.50 ms cm^∓1^. Plants were irrigated and fertigated as necessary, ensuring consistent substrate moisture levels.

The experiment was a randomised block design, with four replicates and 32 plants per replicate. Traditionally, the initial application was made when the plant was at 10% flowering (BBCH 61–65), and subsequent spraying was performed every 7–10 days (a total of four times). Temperature and relative humidity were measured throughout the experiment (Termio + data logger, Poland). The alternative products used in the experiment are presented in Table 7.

The *Bacillus* spp. strains, Bil-LT1_1, Bil-LT1_2, Cran-LT1_8, and Ling-NOR4_15, identification is presented in [51].

The Thyme EO chemical composition was identified according to [10] and is presented in Table 8. In total, 100.00% of Thyme EO components were identified. Thymol 52.22%, p-cymene 12.37%, γ-terpinene 8.39%, and three dominant compounds were determined in Thyme EO.

### 3.2. Measurements

During experiments we evaluated the following: strawberry plant yield (g/plot), one fruit weight (g/fruit), fruit size (mm/fruit), the number of leaves (unit per plant), crowns (unit per plant), flowers (unit per plant), inflorescences (unit per plant), and the EPPO standards PP1/190(3) [26,52] and PP1/16(3) [53]. On each plot, six plants were selected for measurements of crowns, leaves, inflorescences, and flowers in both experimental years in May [54].

The firmness of the strawberries was measured by a texture analyser FR-5105 (FR-5105 Lutron Fruit Hardness Tester FR-5120, Lutron Electronic Enterprise Co., Ltd., Taipei, Taiwan). For the penetration test, a flat-head stainless cylindrical probe of 6 mm diameter was used.

The content of soluble solids (Brix, %) in the fruits was measured with a digital refractometer Atago PAL-1 (Atago Co., Ltd., Tokyo, Japan). Vitamin C (ascorbic acid) was determined by a titrimetric method using a 2,6-dichlorphenolindophenol sodium salt solution (AOAC, 1990) [55].

### 3.3. Statistical Analysis

The research data was analysed using the MS Excel software developed by Microsoft in the United States. All measurements and analyses were performed using the SAS Enterprise Guide software, version 7.1, developed by SAS Institute Inc. in Cary, NC, USA. The analysis of variance (ANOVA) method and Duncan’s test estimated the significance of differences between treatments at *p* < 0.05.

## Figures and Tables

**Table 1:** Vegetative and generative development of strawberry cv. Elsanta and number of elements per plant.

	Crowns		Leaves		Inflorescences		Flowers		
**2023**									
**Control**	2.40	±0.31	13.35	±1.36	3.65	±0.51	25.40	±4.49	a
**Chemical**	2.55	±0.23	15.35	±1.52	3.95	±0.57	31.65	±5.96	ab
**Bacteria**	2.70	±0.21	14.20	±1.18	4.15	±0.50	33.30	±4.37	b
**Thyme**	2.45	±0.32	12.70	±1.35	3.85	±0.60	27.70	±3.84	ab
**2024**									
**Control**	4.25	±0.74	16.90	±2.34	5.90	±0.19	50.60	±2.57	a
**Chemical**	4.20	±0.90	18.20	±1.68	6.05	±0.64	57.90	±3.32	ab
**Bacteria**	4.55	±0.70	21.55	±2.43	6.90	±0.50	64.35	±4.97	b
**Thyme**	4.15	±0.64	17.50	±3.31	7.00	±0.93	58.50	±2.00	ab
	ns		ns		ns				

Note. The same letter indicates no significant differences between treatments, Duncan’s test (*p* < 0.05). ns—not significant.

**Table 2:** Yield of healthy and rotten strawberry cv. Elsanta fruits, g per plot.

Treatment	First Picking		Second Picking		Third Picking		Total
	Healthy	Rotten	Healthy Rotten		Healthy	Rotten	
**2023**							
**Control**	2525.00 ab	0.00 ± 0.00	2875.00 ab	9.74 ± 1.74	2430.00 ab	0.00 ± 0.00	7839.74
**Chemical**	1723.00 a	0.00 ± 0.00	2930.00 ab	11.98 ± 3.00	2600.00 ab	0.00 ± 0.00	7264.98
**Bacteria**	3395.00 bc	0.00 ± 0.00	3305.00 bc	13.57 ± 3.39	2870.00 b	0.00 ± 0.00	9583.57
**Thyme**	2665.00 ab	0.00 ± 0.00	2650.00 ab	8.85 ± 2.21	2205.00 ab	0.00 ± 0.00	7528.85
**2024**							
**Control**	2056.00 a	0.00 ± 0.00	5515.00 ab	70.00 ± 2.00	7630.00 ab	70.00 ± 1.00	14,634.14
**Chemical**	4345.00 bc	0.00 ± 0.00	5250.00 ab	0.00 ± 0.00	7100.00 ab	20.00 ± 0.10	16,695.02
**Bacteria**	3300.00 ab	10.00 ± 0.10	5650.00 ab	0.00 ± 0.00	8844.00 b	30.00 ± 0.10	17,794.04
**Thyme**	4120.00 bc	0.00 ± 0.0	6310.00 b	70.00 ± 1.00	7710.00 ab	170.00 ± 20.00	18,140.23

Note. The average yield of strawberries (g) according to Duncan’s test (*p* < 0.05); the same letter indicates no significant differences between treatments. Means *n* = 4 ± SE.

**Table 3:** Average strawberry cv. Elsanta fruit weight, g.

Treatment	First Picking	Second Picking	Third Picking
**2023**			
**Control**	6.76 ± 0.71	6.73 ± 0.32	5.15 ± 0.53 a
**Chemical**	7.39 ± 0.45	8.28 ± 0.37	7.14 ± 0.42 b
**Bacteria**	8.19 ± 0.28	7.35 ± 0.28	6.60 ± 0.34 ab
**Thyme**	7.05 ± 0.53	6.34 ± 1.01	5.99 ± 0.44 ab
	ns	ns	
**2024**			
**Control**	11.44 ± 0.72	9.78 ± 0.43 a	6.36 ± 0.30
**Chemical**	12.97 ± 0.88	10.42 ± 0.69 a	6.68 ± 0.30
**Bacteria**	13.93 ± 1.00	10.72 ± 1.07 a	7.29 ± 0.52
**Thyme**	12.60 ± 1.18	15.53 ± 4.83 b	6.40 ± 0.65
	ns		ns

Note. The average weight of strawberries (g) according to Duncan’s test (*p* < 0.05); the same letter indicates no significant differences between treatments. Means *n* = 4 ± SE.

**Table 4:** Average fruit diameter of strawberry cv. Elsanta, mm.

Treatment	First Picking	Second Picking	Third Picking
**2023**			
**Control**	23.78 ± 1.58 a	25.05 ± 0.59	24.05 ± 0.19
**Chemical**	25.68 ± 0.56 ab	30.03 ± 1.88	24.75 ± 0.78
**Bacteria**	26.75 ± 0.72 b	29.78 ± 1.13	24.35 ± 0.54
**Thyme**	25.30 ± 0.74 ab	24.53 ± 2.32	23.30 ± 0.52
		ns	ns
**2024**			
**Control**	29.78 ± 1.18	28.55 ± 0.75	24.70 ± 0.83
**Chemical**	31.43 ± 1.43	30.18 ± 0.79	23.50 ± 0.47
**Bacteria**	33.53 ± 1.78	33.93 ± 3.09	24.75 ± 1.26
**Thyme**	30.65 ± 1.81	31.30 ± 0.94	24.55 ± 1.08
	ns	ns	ns

Note. The average weight of strawberries (g) according to Duncan’s test (*p* < 0.05); the same letter indicates no significant differences between treatments. Means *n* = 4 ± SE. ns—not significant.

**Table 5:** Firmness of strawberry cv. Elsanta fruit, N cm^−2^.

Treatment	First Picking	Second Picking	Third Picking
**2023**			
**Control**	8.44 ± 0.71	11.98 ± 0.74	11.22 ± 0.27 ab
**Chemical**	8.46 ± 0.57	12.07 ± 0.47	12.81 ± 0.45 ab
**Bacteria**	9.84 ± 0.62	11.44 ± 1.00	11.36 ± 0.43 ab
**Thyme**	8.34 ± 0.49	11.43 ± 0.74	10.92 ± 0.60 a
	ns	ns	
**2024**			
**Control**	10.49 ± 0.58	11.64 ± 0.56	15.31 ± 1.32 b
**Chemical**	10.61 ± 1.62	12.14 ± 0.80	12.58 ± 0.51 a
**Bacteria**	11.23 ± 0.21	10.59 ± 0.88	11.33 ± 0.55 a
**Thyme**	10.29 ± 0.49	11.64 ± 0.62	11.22 ± 0.54 a
	ns	ns	

Note. According to Duncan’s test (*p* < 0.05), the same letter indicates no significant differences between treatments. Means *n* = 4 ± SE. ns—not significant.

**Table 6:** Content of soluble solids in strawberry cv. Elsanta fruits, Brix %.

Treatment	First Picking	Second Picking	Third Picking
**2023**			
**Control**	9.40 ± 0.20	11.25 ± 0.24	11.03 ± 0.58 b
**Chemical**	9.23 ± 0.13	10.78 ± 0.43	9.50 ± 0.44 a
**Bacterial**	8.45 ± 0.39	10.78 ± 0.27	11.08 ± 0.41 b
**Thyme**	9.40 ± 0.25	10.43 ± 0.40	11.40 ± 0.37 b
	ns	ns	
**2024**			
**Control**	6.69 ± 0.26 a	8.11 ± 0.36	7.48 ± 0.46
**Chemical**	7.36 ± 0.57 ab	6.75 ± 0.46	8.39 ± 0.67
**Bacterial**	7.99± 0.51 b	7.05 ± 0.64	7.44 ± 0.95
**Thyme**	7.53 ± 0.52 ab	6.94 ± 0.60	6.68 ± 0.27
		ns	ns

Note. According to Duncan’s test (*p* < 0.05), the same letter indicates no significant differences between treatments. Means *n* = 4 ± SE. ns—not significant.

**Table 7:** Products used in the experiments.

Treatment	Composition	Dose
Control	None	None, not treated
Chemical	Boscalid 267 g l^−1^ + pyraclostrobin 37 g L^−1^	1.8 kg ha^−1^
	Ciprodinil 375 g l^−1^ + fludioksonil 250 g L^−1^	1.0 kg ha^−1^
Bacteria *	*B. halotolerans* Bil-LT1_1 *B. halotolerans* Bil-LT1_2 *B. velezensis* Cran-LT_1_8 *B. velezensis* Ling-NOR_4_15	3.3 × 10^−6^
Thyme	*Thymus vulgaris*	600 µL/L

* BioSamples in NCBI database: SAMN36292479-SAMN36292482.

**Table 8:** The composition of the essential oil compounds of Thyme (*T. vulgaris*).

Essential Oils	*Thymus vulgaris*	
Component	PA ^1^ (%)	RT ^2^
α-thujene	0.54	5.293
α-pinene	0.83	5.449
Camphene	0.37	5.800
β-pinene	0.22	6.501
1-octen-3-ol	1.57	6.677
Myrcene	1.79	6.895
3-octanol	0.20	7.119
α-phellandrene	0.25	7.241
α-terpinene	1.59	7.572
p-cymene	12.37	7.865
Limonene	0.28	7.935
Eucalyptol	1.72	7.984
γ-terpinene	8.39	8.798
cis-sabinene hydrate	1.04	9.036
Linalool	4.26	10.010
Camphor	0.46	11.442
Borneol	1.24	11.913
Ε-caryophyllene	1.16	19.000
Terpinen-4-ol	1.39	12.233
α-terpineol	0.31	12.900
Thymol methyl ether	0.52	13.842
Thymol	52.22	16.045
Carvacyl methy ether	0.58	14.110
Carvacrol	3.38	16.162
Caryophyllene oxide	0.68	23.179
Other ^3^	2.64	
Total Identified	100.00	

^1^ PA—peak area. ^2^ RT—retention time. ^3^ The compounds that were less than 0.19% of the quantity of the essential oil.

## Data Availability

The data presented in this study are available upon request from the corresponding author.
